# Transgenic Zebrafish Recapitulating *tbx16* Gene Early Developmental Expression

**DOI:** 10.1371/journal.pone.0021559

**Published:** 2011-06-24

**Authors:** Simon Wells, Svanhild Nornes, Michael Lardelli

**Affiliations:** Department of Molecular Biosciences, Special Research Centre for the Molecular Genetics of Development, University of Adelaide, Adelaide, South Australia, Australia; Laboratoire Arago, France

## Abstract

We describe the creation of a transgenic zebrafish expressing GFP driven by a 7.5 kb promoter region of the *tbx16* gene. This promoter segment is sufficient to recapitulate early embryonic expression of endogenous *tbx16* in the presomitic mesoderm, the polster and, subsequently, in the hatching gland. Expression of GFP in the transgenic lines later in development diverges to some extent from endogenous *tbx16* expression with the serendipitous result that one line expresses GFP specifically in commissural primary ascending (CoPA) interneurons of the developing spinal cord. Using this line we demonstrate that the gene *mafba (valentino)* is expressed in CoPA interneurons.

## Introduction

The production of transgenic organisms for research purposes has been a powerful method for accelerating the study of cell lineages in embryo development. This fact in combination with the genetic amenability and accessibility of zebrafish (*Danio rerio*) has stimulated production of an increased number of germline transgenic fish stocks available for such investigations. In particular, using zebrafish promoters linked to green fluorescent protein (GFP) has enabled recapitulation of the expression of various genes and the tracing of cells in embryos as they develop [1], [2], [3].

The zebrafish *spadetail* (*spt*) mutation was first discovered in a screen for recessive mutations affecting neuronal development [4] The gene affected by the *spt* mutation (*tbx16*) was subsequently found to be required for normal morphogenetic cell movement during mesoderm development [5]. The disruption to early development caused by the *spt* mutation results in a lack of trunk mesoderm with cells normally directed to this region accumulating to create a mass of cells at the distal end of the extending tail, the “spade" structure characteristic of these mutants [6], [7].

*tbx16* encodes a member of the T-box family of transcription factors [8], [9]. *tbx16* expression is found in presomitic paraxial mesoderm, the polster and hatching gland cells [9] and a subset of spinal cord cells (first reported by Ruvinsky et. al. (1998) but mistakenly identified as Rohon-Beard neurons). We later demonstrated that these cells are dorsal longitudinal ascending (DoLA) interneurons [10].

DoLAs have a seemingly irregular distribution along the rostrocaudal axis of the spinal cord, a pattern that is particularly difficult to dissect. We have recently shown that there is an underlying cryptic organisation to the rostrocausal and contralateral distribution of these cells [11]. Furthermore, we have shown that this distribution is, to some extent, created by the rostralwards migration of these cells shortly after their birth in the developing spinal cord [11]. Subsequently, we endeavoured to use these neurons to examine the molecules and mechanisms that establish irregular distributions of cells along the rostrocaudal axis in the spinal cord. The creation of a GFP transgenic zebrafish line under the control of the *tbx16* promoter might allow us to examine aspects of DoLA neuron development and migration and examine paraxial mesoderm development in real time.

In this paper we describe an attempt to track the developmental expression of *tbx16*-expressing cells using a transgene possessing 7.5 kb of DNA sequence surrounding the site of transcription initiation of the *tbx16* gene. Four transgenic lines of fish all displayed expression of GFP in the hatching gland progenitors, presomitic mesoderm, newly formed somites, and the hatching gland similar to endogenous *tbx16*. However no expression was observed in DoLA interneurons. Surprisingly one transgenic line of fish expressed GFP in commissural primary ascending (CoPA) interneurons and these cells are shown to be marked by transcripts of the gene *v-maf musculoaponeurotic fibrosarcoma oncogene family, protein B-avian (mafba)/valentino*.

## Materials and Methods

### Ethics Statement

The work was carried out under the auspices of the Animal Ethics Committee and the Institutional Biosafety Committee of the University of Adelaide (Permit number S-033-2006).

### Embryos and staging

Zebrafish were maintained as described [12]. Embryos were collected and allowed to develop at 28.5°C to the required stage. Morphological features of embryos were consistent with the zebrafish staging guide [13].

### Generation of *tbx16*: GFP transgenic lines

We obtained *tbx16* genomic DNA by screening a zebrafish BAC construct library (Genome Systems, Inc., St. Louis, MO, USA) using a *tbx16* cDNA probe. The BAC clone was analysed by Southern blot, and an *EcoRI*-*BamHI* fragment containing 5 kb of 5′ promoter DNA, the first exon and part of the first intron was cloned in frame upstream of GFP in the vector pEGFP-N1 (Clontech, Mountain View, CA, USA). The region for injection was excised from vector sequence, gel purified and was microinjected into the cytoplasm of embryos at the 1-cell stage. GFP expression was analysed before 24 hpf by observation under a fluorescence dissection microscope and embryos positive for GFP expression were raised to sexual maturity. Germline transgenic founders were identified by screening their F1 progeny for GFP fluorescence. Four founders (192A, 512B, 812A, 812C) were isolated, mated with wild-type fish, and their GFP positive offspring were raised to adulthood.

Detailed investigation of developing embryos by fluorescence microscopy was undertaken using a Zeiss Axioplan 2 deconvolution microscope with an AxioCam MRm camera (Carl Zeiss Jena GmbH, Jena, Germany).

### Whole mount *in-situ* transcript hybridisation

The clones for production of probes against *tbx16* and *mafba* transcripts have been described previously [10]. The GFP sequence was excised from pIRES2-EGFP (Clontech) and subcloned into pBluescript (Stratagene Products Division, La Jolla, CA, USA). The insert containing regions from these plasmids were amplified by PCR with M13 primers and then transcribed with T7 or T3 RNA polymerases to produce digoxigenin- (Sigma-Aldrich Corp., St. Louis, MO, USA) or fluorescein- (Sigma) labelled antisense riboprobes. Whole mount *in situ* transcript hybridisation was carried out essentially as described [14] but the two-colour staining reactions were undertaken initially with BCIP/NBT (F. Hoffmann-La Roche Ltd, Basel, Switzerland), and subsequently, the second staining reaction used the Vector Red Alkaline Phosphatase Substrate Kit I (SK-5100, Vector Laboratories Inc., Burlingame, CA, USA). Inactivation of the first alkaline phosphatase reaction was achieved by heating to 65°C for 1 hour in phosphate buffered saline. Older embryos were treated in proteinase K as described [14]. Light field observations were conducted under a Zeiss Axiophot microscope (Carl Zeiss) with a 20× objective using differential interference contrast (DIC) optics.

## Results

### Generation of *tbx16*:GFP transgenic zebrafish

**Figure 1:**
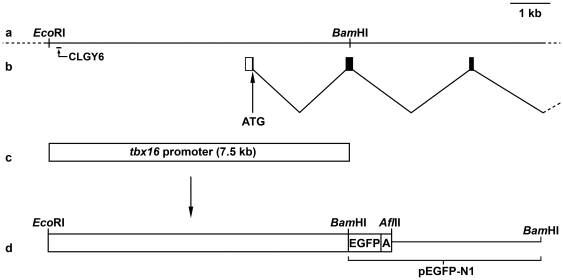
Genomic region of the *tbx16* gene and transgenic construct. (a) *Eco*RI and *Bam*HI restriction sites in the genomic region of the *tbx16* gene surrounding the transcription initiation site. The interval marked “CLGY6" indicates the published enhancer trap insertion site resulting in GFP expression that recapitulates *tbx16* gene expression [15]. (b) Boxes indicate the first three exons and filled boxes are protein-coding regions. The start of translation is shown (ATG). (c) The *Eco*RI/*Bam*HI fragment of the *tbx16* promoter used in the transgenic construct includes the translation start site, the first exon, and part of the second exon of the *tbx16* gene. (d) The promoter fragment was cloned in-frame upstream of enhanced GFP (EGFP) and a SV40 polyadenylation signal (A) in the multiple cloning site of the pEGFP-N1 vector.

We have previously shown that *tbx16*-expressing neurons are migratory and this migration contributes to their distribution in the developing spinal cord [11]. To examine further these cells including their origins and interaction with other cells of the spinal cord we attempted to label them with GFP by creating transgenic fish expressing GFP under the control of the *tbx16* promoter. A 7.5 kb genomic DNA fragment including 5 kb of 5′ promoter (Fig. 1a) and 2.5 kb of transcribed *tbx16* gene sequence (Fig. 1b) – the “*tbx16* promoter" (Fig. 1c) was cloned upstream of EGFP in the pEGFP-N1 vector (Fig. 1d, see Materials and Methods). This promoter region included the site of insertion in the enhancer trap line CLGY6 that was shown to recapitulate *tbx16* expression at 24 hpf [15]. Independent lines of transgenic zebrafish were generated by injecting linearised construct into single-cell embryos. Germline transmission was found in 2.6% of examined surviving adults (not all mosaic adults were screened). Four founders (192A, 512B, 812A, 812C) were isolated, mated with wild-type fish, and their GFP positive offspring were raised to adulthood. Founder germline mosaicism was determined to be between 4 and 40%. To confirm germline integration of the transgene, heterozygous GFP expressing F1 embryos were raised to sexual maturity and outbred to wild-type fish. Transgenic F2 progeny were generated at approximately 50% (49–52%, n>870 for each line) indicating a single insertion site in each founder, and a typical Mendelian inheritance pattern. Each line has consistently retained its pattern of GFP expression for at least 3 generations.

### Consistent expression of *tbx16*:GFP in embryonic zebrafish

**Figure 2:**
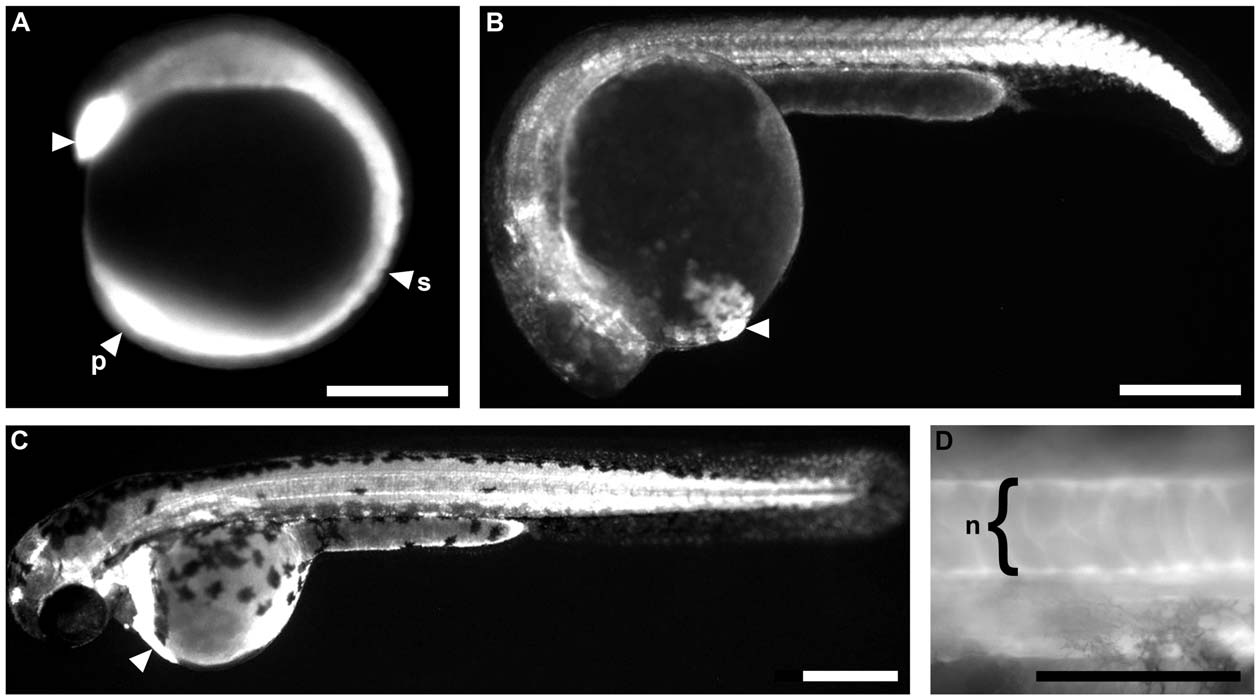
GFP expression in stable transgenic lines consistent with *tbx16* expression in wild type embryos. (a) At 12 hpf strong expression is seen in the polster (arrowhead), and the presomitic mesoderm (p). Expression persists in mesoderm that has formed somites (s). (b) At 24 hpf GFP expression is seen in the hatching gland (arrowhead) and in posterior somites, while it has faded from the earliest formed somites. (c) At 48 hpf expression remains in the hatching gland that has formed from the polster (arrowhead) and persists in the posterior somites. (d) The same embryo as in (c). GFP expression is observed throughout the notochord (n) highlighting cell extremities. Lateral views of embryos, anterior is left and dorsal is up. Scale bars in a–c = 250 µm, in d = 100 µm.

We examined transgenic lines for temporal and spatial expression of GFP. All examined transgenic lines displayed extensive GFP fluorescence consistent with the expression of *tbx16* transcript in wild type embryos. Transgene expression was first detected at 12 hpf in the hatching gland progenitors, presomitic mesoderm and the tail bud (Fig. 2a). GFP expression at this stage was also noted throughout the somitic mesoderm indicating that the protein persists longer in the developing mesoderm than endogenous *tbx16* transcripts. Expression at 24 hpf is seen in the hatching gland and in the posterior somites while expression has faded from the earliest formed somites (Fig. 2b). By 48 hpf GFP expression in somitic mesoderm has diminished in all lines except 192A, allowing cells of the spinal cord to be viewed (Fig. 2c). It is unknown when spinal cord neurons commence expression of GFP due to the masking effect of overlying somitic mesoderm obscuring more medial tissues. At 48 hpf all lines strongly express GFP in the hatching gland. Peculiarly, all lines show some level of expression in the notochord (Fig. 2d). Line 192A displays persistent low-level somitic mesodermal expression at 96 hpf (not shown).

### Tissue specific GFP fluorescence patterns

**Figure 3:**
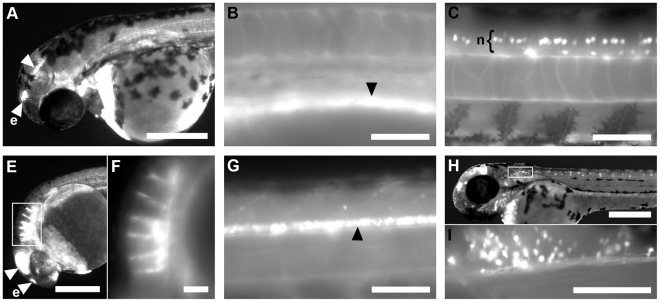
Expression unique to individual stable transgenic lines. (a) Line 192A at 48 hpf. GFP expression is observed in regions of the midbrain (arrowhead) and in the epiphysis (e). Line 512B at 24 hpf (b) and 48 hpf (c). Expression is seen in the dorsal aorta and in ventral neurons (n) of the spinal cord. Line 812A at 24 hpf (d–e) and at 48 hpf (f). Expression can be seen in the epiphysis, the midbrain and in a banding pattern in rhombomeres (boxed). White box indicates the area enlarged in (e). Later expression is observed in the floor plate of the spinal cord (f). (g–h) Line 812C at 48 hpf. Expression is seen in the epiphysis and the midbrain. GFP is also noted in a specific subpopulation of spinal cord neurons and neurons posterior of the otic vesicle in the hindbrain (boxed). White box indicates the area enlarged in (h). Lateral views of embryos, anterior is left and dorsal is up. Scale bars in a, d, g = 250 µm, in b, c, e, f, h = 50 µm.

The earliest GFP expression appears to be consistent among the lines, with line-specific expression beginning later in development. At 24 hpf line 192A displays GFP expression in cells in the midbrain (not shown). At 48 hpf (and 96 hpf) this expression pattern has coalesced into two distinct regions that appear to consist of distinct cells in the midbrain and the epiphysis (Fig. 3a). At 24 hpf line 512B expresses GFP in the dorsal aorta (Fig. 3b) and later at 48 hpf in ventrally projecting neurons in the ventral spinal cord (Fig. 3c). These neural cells persist until at least 96 hpf (not shown). 24 hpf embryos of the 812A line have a large portion of the midbrain expressing GFP along with the epiphysis and show a distinct banding pattern in the hindbrain (Fig. 3d) that appears to be related to the rhombomeres (Fig. 3e). This pattern disappears by 48 hpf. At 96 hpf the line 812A expresses GFP in the floor plate of the spinal cord (Fig. 3f). In line 812C at 48 hpf we observe expression of GFP in the epiphysis and regions of the midbrain. This line also shows expression in dorsal neurons in the spinal cord in an apparently irregular rostrocaudal distribution that appears similar to that of DoLA interneurons (Fig. 3g). These embryos display expression in distinct neural cells in the hindbrain (Fig. 3h). Tissue specific expression is summarised in Table 1.

**Table 1:** Tissue specific GFP fluorescence patterns.

	24 hpf	48 hpf	96hpf
192A	midbrain	midbrainepiphysis	midbrainepiphysis
512B	dorsal aorta	dorsal aortaventral spinal cord neurons	ventral spinal cord neurons
812A	midbrainepiphysishindbrain/rhombomeres	N/A	floor plate
812C	N/A	epiphysismidbrain neuronsCoPA interneurons	N/A

GFP expression diverges between the transgenic lines at later developmental time points. Expression of GFP is noted in numerous tissues over the first 96 hpf (N/A: not applicable).

### Neurons expressing GFP in the spinal cord of the 812C line are comissural primary ascending interneurons (CoPAs)

**Figure 4:**
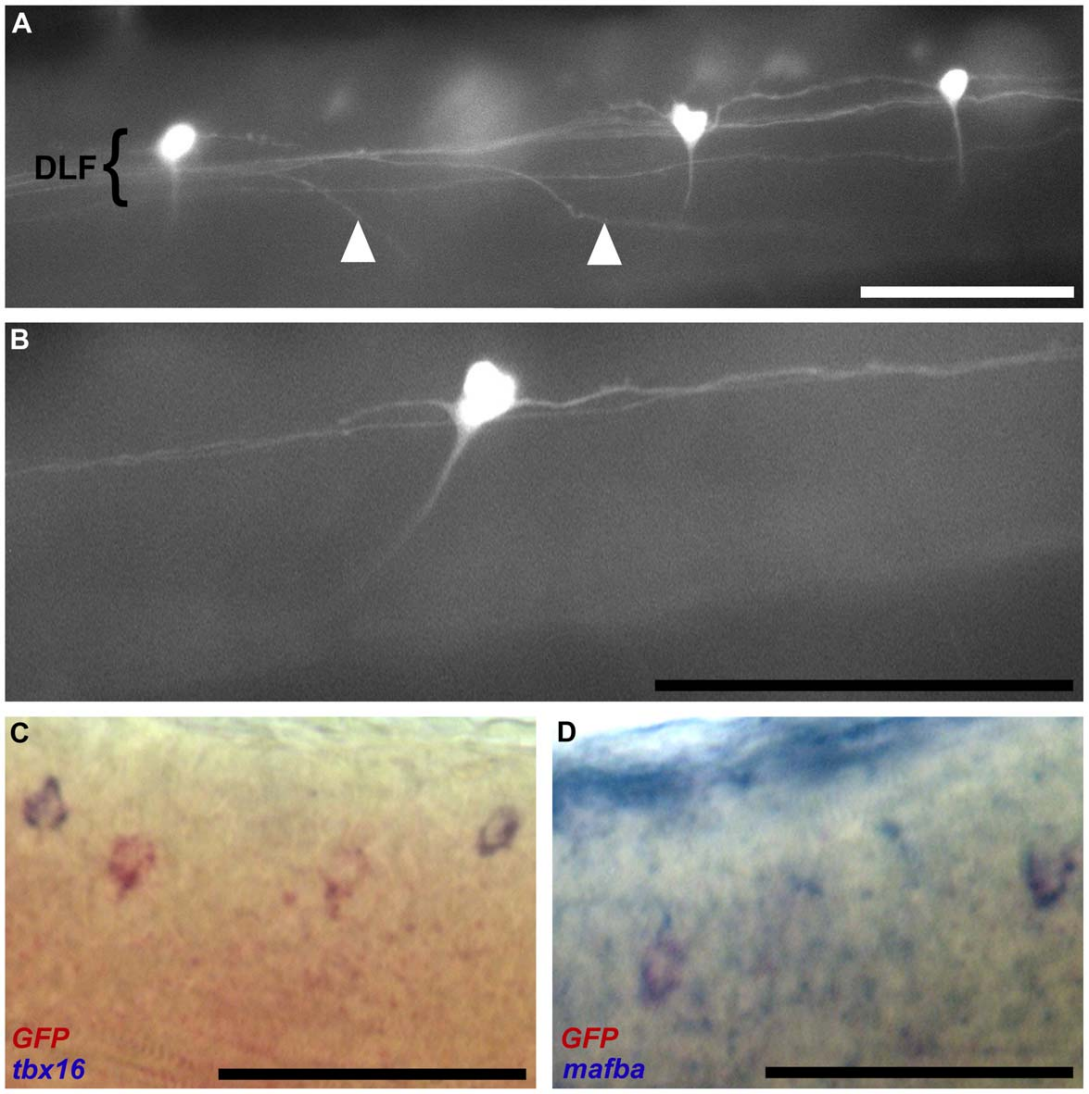
Identification of spinal cord neurons in line 812C at 48 hpf. (a) Lateral view of three GFP-positive neural cells situated in a non-uniform distribution along the dorsal longitudinal fasiculus (DLF) indicated by a tight grouping of neuronal projections. Axons projecting from neurons located in the contralateral DLF can be seen to rise from the ventral midline before joining the in-focus DLF (arrowheads). (b) Lateral view of a single GFP-positive neuron illustrating the projections originating from the cell. Two projections extend within the DLF – one ascending and one descending – and a third ventrally extending axon turns anteriorly and fades from view as it approaches the ventral midline before ascending in the contralateral DLF (not shown). (c) Two-colour *in situ* transcript hybridisation indicating that GFP-positive cells (stained red) are not the *tbx16*-positive dorsal longitudinal ascending interneurons (stained blue). (d) Two-colour *in situ* transcript hybridisation showing that neurons expressing GFP (stained red) also express *mafba* (stained blue). All embryos are positioned anterior left and dorsal up. Scale bars indicate 100 µm.

We have previously shown that the *tbx16*-expressing cells of the spinal cord are the dorsal longitudinal ascending (DoLA) interneurons. GFP positive spinal cord cells observed in the 812C line (Fig. 3g) exhibit characteristics consistent with DoLA cells; both cell types show an apparent irregular rostrocaudal distribution of approximately 20–25 cells per embryo with similar dorsoventral positioning and they occur at similar developmental times. Upon closer examination, the GFP expressing cells have properties differing from the published morphology of DoLA cells [16], [17]. Although the GFP expressing neurons are distributed in an apparently irregular rostrocaudal pattern and can be found along the dorsal longitudinal fasciculus (Fig. 4a) they have ascending and descending longitudinal projections, and a single ventral projecting axon that crosses the midline and ascends in the DLF contralateral to the cell body (Fig. 4a, b). The morphology and distribution of these cells is consistent with them being commissural primary ascending (CoPA) interneurons.

To confirm that the 812C transgenic line is not expressing GFP in the DoLA neurons, we undertook *in situ* transcript hybridisation using probes against *GFP* and *tbx16* simultaneously. This showed that the neurons expressing *GFP* do not simultaneously express *tbx16*, although the two cell types are found at the same dorsoventral level of the developing spinal cord (Fig. 4c). We examined the expression of another transcript, *mafba* known to be expressed in a similar distribution in the developing spinal cord but not in *spt*-expressing DoLA neurons {Tamme, 2002 #8} and discovered that the *mafba*-expressing cells show coexpression of *GFP* (Fig. 4d). Thus expression of *mafba* appears to mark CoPA interneurons.

## Discussion

In this paper we have described an attempt to produce transgenic zebrafish to track the developmental expression of *tbx16* by using GFP fused to *tbx16* promoter sequence.

We used a 7.5 kb segment of genomic DNA that encompassed 5 kb of the 5′ promoter and includes the translation start site, the first exon, and part of the second exon of the *tbx16* gene. Extensive outbreeding has shown that these lines are all single insertion site transgenic lines that show typical Mendelian inheritance.

The four lines of fish examined display expression of GFP in the hatching gland progenitors (the polster), presomitic mesoderm, newly formed somites, and the hatching gland consistent with endogenous *tbx16* expression and perdurance of GFP from expression in progenitor tissues. However, none of the lines express GFP in the DoLA neurons which was the original purpose for which they were created. Later in development expression of GFP in the four lines diverges somewhat from endogenous *tbx16* expression and from each other. Persistent expression of enhanced GFP in lateral tissues can mask expression in deeper medial tissues, suggesting that destabilised GFP might have been a better choice for creating these particular transgenic zebrafish.

The common differences in expression between the transgenic lines and endogenous *tbx16* expression (e.g. absence of expression in DoLAs in the transgenic fish) are most likely due to the absence of some *tbx16* promoter regulatory sequences from the construct. Our construct includes 5 kb of 5′ promoter sequence, an amount that has been shown to be sufficient to replicate the expression patterns of many endogenous genes in transgenic fish [18], [19], [20]. However, the work of Kikuta *et. al.* [21] has shown that genes can be classified into two broad types depending on whether their transcription is regulated by elements close to the transcription initiation site (“bystander genes") or dispersed over relatively large genetic distances defining “genomic regulatory blocks" (GRBs). Typically, the genes regulated by elements dispersed over GRBs are those regulating embryo development such as *tbx16*.

The particular differences in transgene expression between the individual lines are more likely due to the influence upon their transcription of different regulatory elements flanking the transgene insertion sites in the genome. Interestingly, one transgenic line, 812C, uniquely expresses GFP in a subset of spinal interneurons. These cells are distributed in a seemingly irregular pattern and can be found along the dorsal longitudinal fasciculus (Fig. 4a). They have ascending and descending longitudinal projections, and a single ventral projecting axon that crosses the midline and ascends in the DLF contralateral to the cell body (Fig. 4a, b). The morphology and distribution of these cells is consistent with them being commissural primary ascending (CoPA) interneurons. Furthermore these cells have been shown to be marked by transcripts of the *mafba* gene. The 812C transgenic line may be useful for studies of the birth and development of CoPAs and their projections.

The zebrafish spinal cord consists of a limited number of discrete neuronal cell types. Although it is possible to distinguish each of these types by their spatiotemporal position and morphology few unique molecular markers of these cells have been discovered. Circumferential Ascending (CiA) interneurons express *Eng1b* transcripts [22]. *alx* is expressed in Circumferential Descending (CiD) interneurons although it appears, perhaps, that not all CiD neurons express this gene [23]. We previously showed that DoLA cells express *tbx16* [10]. We believe that the gene *mafba* – the expression pattern of which superficially resembles that of *tbx16* that marks DoLAs in the spinal cord - may be a unique marker of another zebrafish spinal neuron, the CoPA interneuron. Since DoLAs and CoPAs share very similar dorsoventral positions and rostrocaudal distributions in the spinal cord, it is interesting to speculate that regulatory elements within our transgene are cooperating with elements flanking the transgene insertion site to alter activation of transcription away from DoLAs so that it occurs instead in CoPAs. Identification of the GRB in which our 812C insertion has occurred may help to explain this phenomenon. However, the idea that some elements required for neuronal expression occur within the 7.5 kb of DNA from the *tbx16* locus within our transgene is supported by the fact that all of our transgenic lines show expression at some stage in neurons.
